# Role of Intestinal Microbes in Chronic Liver Diseases

**DOI:** 10.3390/ijms232012661

**Published:** 2022-10-21

**Authors:** Mengyi Xu, Kangkang Luo, Junjie Li, Yu Li, Yuxuan Zhang, Zhiyao Yuan, Qiang Xu, Xudong Wu

**Affiliations:** 1 State Key Laboratory of Pharmaceutical Biotechnology, School of Life Sciences, Nanjing University, Nanjing 210023, China; 2 Nanjing Stomatological Hospital, Medical School of Nanjing University, Nanjing 210008, China

**Keywords:** intestinal microbes, bacterial metabolites, chronic liver diseases, probiotics and prebiotics

## Abstract

With the recent availability and upgrading of many emerging intestinal microbes sequencing technologies, our research on intestinal microbes is changing rapidly. A variety of investigations have found that intestinal microbes are essential for immune system regulation and energy metabolism homeostasis, which impacts many critical organs. The liver is the first organ to be traversed by the intestinal portal vein, and there is a strong bidirectional link between the liver and intestine. Many intestinal factors, such as intestinal microbes, bacterial composition, and intestinal bacterial metabolites, are deeply involved in liver homeostasis. Intestinal microbial dysbiosis and increased intestinal permeability are associated with the pathogenesis of many chronic liver diseases, such as alcoholic fatty liver disease (AFLD), non-alcoholic fatty liver disease (NAFLD), non-alcoholic steatohepatitis (NASH), chronic hepatitis B (CHB), chronic hepatitis C (CHC), autoimmune liver disease (AIH) and the development of hepatocellular carcinoma (HCC). Intestinal permeability and dysbacteriosis often lead to Lipopolysaccharide (LPS) and metabolites entering in serum. Then, Toll-like receptors activation in the liver induces the exposure of the intestine and liver to many small molecules with pro-inflammatory properties. And all of these eventually result in various liver diseases. In this paper, we have discussed the current evidence on the role of various intestinal microbes in different chronic liver diseases. As well as potential new therapeutic approaches are proposed in this review, such as antibiotics, probiotics, and prebiotics, which may have an improvement in liver diseases.

## 1. Introduction

Epidemiological, physiological, and histological datasets suggest that microbial communities play a considerable role in regulating human health [1,2,3]. The microbial populations residing in the human intestine are collectively referred to as microbes, and their genetic pool is an order of magnitude higher than the human genome. At present, in addition to 16s rRNA sequencing, the emerging metagenomics sequencing uses the novel high-throughput sequencing (HTS) technology to investigate the genomes of communities of microorganisms in specific environments, and to analyze differences in microbial diversity, population structure, and evolutionary relationships [4]. It can further explore the functional activity of microbial communities, interoperative relationships, and relationships with the environment [5]. These datasets can reveal many associations between individual microbial species or communities and various diseases.

There is growing evidence of a significant role for intestinal microbes in human diseases, interacting with many extra-intestinal organs such as kidneys, brain, cardiovascular and skeletal systems [6]. There is an inextricable two-way link between the liver and intestine, with bile and its metabolites being transported from the liver to the intestine via the bile duct, while microorganisms in the intestine use food residues and bile as substrates, producing several products that are transported back to the liver via the portal vein [7]. Therefore, researchers have focused on the interactions between intestinal microbes and liver diseases, and have pulled back the curtain on how intestinal microbes and their associated metabolites influence liver homeostasis and disease progression. In recent years, numerous studies on intestinal microbes affecting the metabolic and immune functions of the liver. It has been verified that the composition and dysbiosis of microbes in the intestinal may be closely linked to the pathogenesis of many liver-accruing diseases, such as chronic hepatitis, alcoholic fatty liver disease (AFLD), non-alcoholic fatty liver disease (NAFLD), non-alcoholic steatohepatitis (NASH), cirrhosis, and hepatocellular carcinoma (HCC) [8,9,10,11,12].

A few specific microbes associated with the pathogenicity or severity of liver diseases have been reported. But the underlying mechanisms have been unclear or have shown only the tip of the entire iceberg. For example, antibiotics pretreatment could attenuate liver transplant injury in mice and humans [13]. However, antibiotics sometimes showed contradicting effects that antibiotics reduced microbial diversity that led to some increased acute liver injury, as well as the extent of liver damage in AIH mice [14]. Besides, antibiotics-mediated inhibition of tumor progression in HCC had different interpretations [15]. It is because a wide range of factors is involved in intestine-mediated hepatic effects, and multiple signaling pathways regulate the interactions of intestinal microbes and the liver.

**Figure 1:**
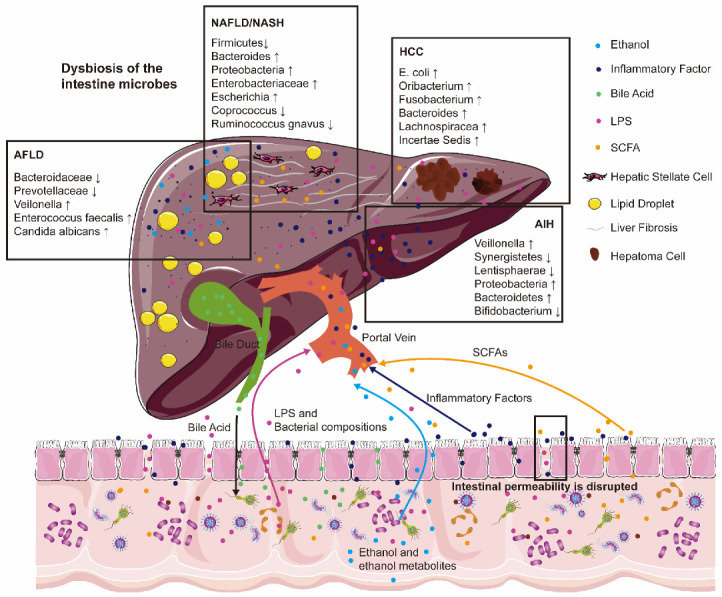
Intestinal microbial dysbiosis and increased intestinal permeability are associated with the pathogenesis of many chronic liver diseases. Bacterial compositions (lipopolysaccharides (LPS)), intestinal bacterial metabolites (ethanol and ethanol metabolites, short-chain fatty acids (SCFAs)), and inflammatory factors are deeply involved in alcoholic fatty liver disease (AFLD), non-alcoholic fatty liver disease (NAFLD), non-alcoholic steatohepatitis (NASH), autoimmune liver disease (AIH) and the development of hepatocellular carcinoma (HCC). Arrows with different colors represent the flow direction of different metabolites between liver and intestine. “↑” represents the increased abundance of the corresponding bacteria. “↓” represents the decreased abundance of the corresponding bacteria.

Previously, several reviews have been published, focusing on the wide world of intestinal microbes. They also presented a section on the contribution of intestinal microbes to the pathophysiology of liver diseases [16,17,18]. Here, we discussed current research on the correlation between intestinal microbes and different liver diseases. Dysbiosis of the intestinal microbes plays an important role in the pathogenesis of chronic liver diseases including AFLD, NAFLD, NASH, CHB, CHC, AIH, HCC, and others (Figure 1). And we also have summarized new approaches to intestinal microbes-based research in the context of existing clinical treatments and drugs. Importantly, prospective technologies have been presented on possible directions in this review, which would be helpful to delve deeper into chronic liver diseases influenced by intestinal microbes.

## 2. Methods

**Figure 2:**
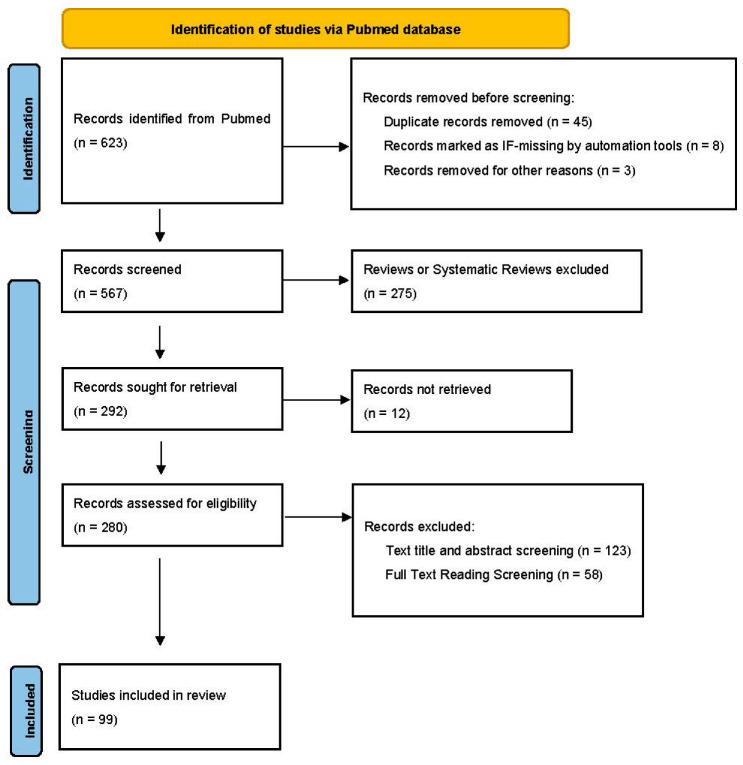
PRISMA workflow for review, which illustrates the relationship between intestinal microbes and chronic liver diseases.

We systematically searched papers using the PRISMA method (Figure 2) [16]. Above all, we searched and collected papers related to intestinal microbes and different chronic liver diseases from the PubMed database, by using different keywords. After the first screening, we excluded duplicate papers and papers without impact factor. Papers written in non-English languages were also excluded. In the second screening, papers in the reviews or systematic reviews category were removed and we focused on research papers. And then, we read the titles and abstracts of the manuscript and removed articles with low relevance to the central elements of this review. Finally, we selected some high-quality articles for summarization and description by systematical learning of the full text. When we used this method to screen the references, there was a certain researcher bias.

## 3. Influence of Intestinal Microbes on the Intestinal Functions

There are many ways in which intestinal microbes can affect human health, such as released vesicles, bacterial components, and metabolites of intestinal microbes [17]. The intestinal microbes are colonized by over 100 trillion individuals in the human intestine [18]. Advances in molecular biology have now made it possible to analyze intestinal microbes at the DNA and RNA levels without culture. At the same time, next-generation sequencers have been developed, for example, 16S ribosomal RNA-targeted analysis and metagenomic sequencing analysis [19,20]. It has been found that the core microorganisms in the intestinal consist of five species: 79.4% phyla-Firmicutes, (*Ruminococcus*, *Clostridium*, and *Eubacteria*), 16.9% Bacteroidetes (*Porphyromonas*, *Prevotella*), 2.5% Actinobacteria (*Bifidobacterium*), 1% Proteobacteria and 0.1% Verrumicrobia [21]. In addition, *Lactobacilli*, *Streptococci*, and *Escherichia coli* are also found in the intestinal tract [21].

The microbial composition is influenced by many endogenous and exogenous factors, such as host physiology and immunity, diet, and medication [22]. The alteration of microbes in our environment and the dysbiosis of intestinal microbes are mainly derived from the following factors: excessive hygienic lifestyle, minimal exposure to environmental microbes, sophisticated food processing, frequent use of antibiotics, etc. [23]. Powerful perturbations mentioned above can lead to a state of ecological dysregulation, which is associated with many human diseases.

Balanced microbes play a role in maintaining the intestinal barrier, which is essential for the in vivo homeostasis and function of the intestinal [24]. While, dysbiosis of the microbes always damaged the intestinal epithelial barrier and led to the so-called leaky intestine, which brought the intestinal contents into the body’s internal environment and could induce inflammatory reactions which even induce serious sepsis [25]. In the leaky intestine, disruption of the barrier could lead to the systemic spread of microorganisms and entry into the liver circulation [26]. In addition, the lymphatic system is highly enriched in the mucosa outside the intestine. Dysbiosis of microorganisms could affect the mesenteric lymphatic system and eventually enter the body’s circulation, provoking an immune response in the gastrointestinal tract and the whole body [27]. Moreover, the intestine is also innervated by hundreds of millions of neurons, and signals are transmitted along the intestinal neurons to various target organs [28].

Dysbiosis of the microbes could induce intestinal inflammation, which conversely exacerbated intestinal microbial dysbiosis, ultimately creating a vicious cycle. For example, elevated levels of LPS, which were highly pro-inflammatory in the bacterial wall, would induce intestinal inflammation. The subsequent oxidative stress induced by LPS would in turn promote the proliferation of facultative anaerobes and exacerbate the dysbiosis of the intestinal microbes [29]. It has been found that as the relative proportion of facultative anaerobes increases and the anaerobes which have protective functions decrease, certain SCFAs production also decreases [30]. SCFAs were well known to promote intestinal cell regeneration and maintain the intestinal barrier, and they also showed anti-inflammatory properties [31].

The regulation of intestinal microbial ecology and the enhancement of intestinal epithelium integrity are both essential for the long-term restoration of intestinal homeostasis. Several microbiological therapies were thought to enhance or restore the integrity of the intestinal barrier [32,33,34]. Although no clinical drugs have yet been approved, most of them show effective mechanisms of action, which have promising applications in the protection of the intestinal barrier. The main probiotics that have been the focus of research are *Lactobacillus*, *Bifidobacterium*, and *Saccharomyces* [35]. While their mechanisms of action have not been elucidated, some evidence suggests that probiotics may be involved in the healing of intestinal epithelial damage in diverse ways. *Lactobacillus plantarum* MB452 increases the expression of tight junction-related genes in the intestinal epithelium of human colorectal adenocarcinoma cells (Caco-2) [36]. Furthermore, two soluble proteins in *Lactobacillus* GG fermented milk supernatant, P40, and P75, not only activated epidermal growth factor receptors to promote the survival and growth of intestinal epithelial cells but also prevented cytokine-induced apoptosis [37]. Prebiotics (certain SCFAs) are defined as selectively fermented component that results in specific changes in the composition or activity of the gastrointestinal microbiota that are beneficial to the health of the host [38]. *Eubacterium rectale*, *Clostridium coccoides*, and *Roseburia* were essential bacteria to produce butyrate, which provided nutrients to colon cells [39]. Individual SCFAs were associated with increased mucus secretion from the intestinal surface of mice, maintenance of immune homeostasis, and induction of T-reg lymphocytes [40]. Insulin was also a prebiotic that may protect human colon cells by reducing LPS [41].

The intestinal microbes can regulate intestinal permeability positively or negatively through metabolic and immune pathways. There is mounting evidence to support an etiological link between intestinal permeability and extraintestinal diseases, with the strongest link to liver diseases. Therefore, mechanistic studies and therapeutic approaches to liver diseases with a focus on the intestine need to be investigated for further research.

## 4. Intestinal Microbes and Non-Alcoholic Fatty Liver Disease (NAFLD)

NAFLD is now considered to be the most common chronic liver disease, affecting approximately 25% of adults worldwide [42]. Recently, the prevalence of NAFLD has increased dramatically in parallel with the global increase in the number of people with metabolic syndrome, diabetes, and obesity [35]. NAFLD is a continuum of liver disease that includes simple steatosis, NASH, fibrosis, cirrhosis, and HCC [43].

The two-hit theory of the liver was first proposed as a mechanism for the pathogenesis of NAFLD [44]. The first hit was hepatic lipid deposition and insulin resistance, and the second was oxidative stress in the liver [44]. The subsequent studies over the past decade have shown that the pathogenesis of NAFLD/NASH is much more complex than the “two-hit theory”. As a result, the “multiple hit theory” has been progressively proposed, including genetic susceptibility, epigenetic modifications, abnormal glycolipid metabolic signaling pathways in the liver, immune cell attack in the liver, and adipokines secreted by adipocytes [45]. Today, NAFLD has been a systemic metabolic disorder. Increasing evidence suggested that metabolic disorders, caused by intestinal microbial dysregulation, also played an important role in the pathogenesis of NAFLD [46,47]. Endotoxaemia caused by increased intestinal permeability has been observed in patients with NAFLD, suggesting that intestinal permeability-induced inflammatory pathways contributed to the pathogenesis of NAFLD [48]. The intestinal microbes were affected even in the early stages of NAFLD, and intestinal microbial homeostasis became more unstable as the disease progresses to more advanced stages. The intestinal microbes of patients with advanced liver disease and cirrhosis were characterized by an increase in potentially pathogenic bacteria and a decrease in the number of beneficial bacteria in the intestine [49,50].

Extensive investigations have been carried out to identify specific pathogenic species. Both population-based and animal-based studies of the intestinal microbes showed that some microbial populations (*Bacteroides*, *Ruminococcus*, etc.) were significantly altered in NAFLD compared to normal individuals [51]. The abundance of bacteria in the feces of patients with NASH was lower compared to healthy control patients. One study in pediatric patients discovered that Proteobacteria, Enterobacteriaceae, and Escherichia showed significant differences in the microbiome of obese and NASH patients [52]. They also found that Proteobacteria produced significant levels of ethanol, which may be responsible for higher levels of liver damage [52]. Clinical trials have shown that hepatic steatosis is associated with lower intestinal microbial diversity and the presence of *Coprococcus* and *Ruminococcus gnavus* [53]. Loomba et al. found that the intestinal microbes of NASH patients also differed in early and late liver fibrosis. They could determine advanced liver fibrosis based on the increase of Proteobacteria and the significant decrease of Firmicutes in the intestine of the patients [50]. The severity of NAFLD was closely related to intestinal ecological dysregulation, where the abundance of *Bacteroides* was associated with the development of NASH and the abundance of *Ruminococcus* was implicated in further fibrosis of the liver [51].

In terms of mechanism, intestinal microbes impact hepatic triglyceride metabolic homeostasis by increasing endotoxin levels, affecting nutrient absorption, and altering the type and content of metabolites, such as amino acids, fatty acids, and bile acids in the body [54]. In addition, intestinal microbes could influence the metabolic and inflammatory state of the body and liver through the metabolism of nutrients and the release of anti-inflammatory or pro-inflammatory compounds. The majority of investigations into the association between the intestinal microbes and the pathogenesis of NAFLD have concerning changes in the bacterial polysaccharides synthesized and SCFAs produced in the intestine (such as acetate, propionate, and butyrate) [55]. It has been found that SCFAs specifically bind to G protein-coupled receptors (GPCRs) such as GPR41 and GPR43, which are expressed in adipose tissue, liver, and intestine and induce hepatic lipogenesis, cholesterol synthesis and affect glucose homeostasis [55]. Although SCFAs appear to promote NAFLD either directly or through bound to GPCRs, other effects of SCFAs have also been demonstrated that may be beneficial for NAFLD, mainly through the regulation of adenosine 5‘-monophosphate-activated protein kinase (AMPK) activity in the liver [56]. In addition, butyrate has been shown to affect colonic epithelial regeneration, thus providing an important mechanism for the regulation of intestinal integrity [57]. Propionate may also bind to GPCR43, which is expressed on lymphocytes to maintain the body’s intrinsic immune defenses [58].

Several probiotics have been found to have beneficial therapeutic effects on NAFLD in several recent investigations of animal models of NAFLD. Several probiotics had positive effects on NAFLD liver oxidative stress and inflammatory liver injury mediated by c-Jun N-terminal kinase (JNK) and nuclear factor-κ-gene binding (NF-κB) in mouse models, with improvements associated with insulin resistance [59,60]. Besides experiments with animals, several clinical trials of probiotics for patients with NAFLD have been reported. The main clinical uses for NAFLD were a mixture of beneficial bacteria: *Streptococcus thermophilus*, *Bifidobacterium breve*, *Bifidobacterium longum*, *Bifidobacterium infantis*, *Lactobacillus acidophilus*, *Lactobacillus plantarum*, *Lactobacillus paracasei*, and *Lactobacillus bulgaricus* (VSL #3) [60,61]. It was found that conjugated linoleic acid (CLA), a microbial metabolite produced by the VSL #3 probiotic, was associated with improvements in NAFLD [60,61].

## 5. Intestinal Microbes and Alcoholic Fatty Liver Disease (AFLD)

In a Global Report on Alcohol and Health 2018 published by the World Health Organization, approximately 3 million people die from alcohol consumption globally each year, accounting for 5.3% of all deaths. A large proportion of these deaths are attributed to alcoholic fatty liver disease (AFLD) [62].

AFLD is caused by chronic alcohol abuse. There is no such thing as a safe dose of alcohol and the damage that each sip can do to the body. Clinically, 30% of heavy drinkers will suffer from severe AFLD, NASH, and cirrhosis, or even develop HCC [63]. The liver is the main organ to metabolize alcohol, and the process of ethanol metabolism will cause irreparable damage to the liver. When the body was stimulated by ethanol, the excessive input of lipids led to a disruption of lipoprotein synthesis and metabolism in the liver. Inadequate oxidation of fatty acids in the liver led to the deposition of lipids in the liver, resulting in the development of a fatty liver and inflammation [64,65]. In addition, it has also been found that alcohol affects intestinal permeability, leading to damage to the intestinal epithelium [66]. It also caused dysbiosis of the intestinal microbes, increased susceptibility to liver diseases, and increased liver damage [64].

Intestinal inflammation caused by dysbiosis, acetaldehyde (as a product of ethanol metabolism), alterations in intestinal bile acids, and possibly other metabolites, led to disruption of the intestinal barrier [67]. Ethanol and its metabolite acetaldehyde disrupted the tight junctions of the intestinal epithelium, thereby affecting intestinal permeability. The junctional proteins affected by ethanol include transmembrane proteins (occludin, junctional adhesion molecule-A, and claudins), scaffolding proteins (ZO1), and associated signaling molecules such as myosin light chain kinase (MLCK) and Rho-associated kinase (RHOA) [68,69,70]. Endotoxin levels were significantly elevated in serum samples from patients with AFLD, and alcohol abuse causes transient endotoxemia in healthy subjects [71]. The first organ reached by intestine-derived microbial products and alcohol metabolites is the liver. Several pathogen-associated molecular pattern receptors, such as toll-like receptors (TLRs) and NOD-like receptors (NLRs), on hepatic parenchymal and non-parenchymal cells in the liver could recognize components of gram-positive bacteria including the lipid A fraction of LPS, flagellin and bacterial DNA [72]. There was further activation of Kupffer cells and infiltration of macrophages which can produce various inflammatory cytokines and chemokines causing more severe liver damage [72].

The intestinal microbes found in numerous studies are confirmed to be essential factors in the progression of AFLD. A study has collected intestinal microbes from AFLD patients and normal humans to colonize the intestine of mice in order to construct humanized mouse models [73]. Liver damage was significantly more severe in mice colonized with microbes from AFLD patients under ethanol induction compared to control mice colonized with microbes from normal humans [73]. Clinical trials have also found a significant increase in clinical survival in patients with severe AFLD after microbial transplantation via intranasal catheters [74]. In addition, it was demonstrated that the proportion of Bacteroidaceae and Prevotellaceae in the intestine is lower in patients with AFLD [75]. Clinical findings suggested that patients with AFLD showed an increased proportion of specific strains of bacteria in the intestine, including *Veilonella* and *Enterococcus faecalis* [76]. *Enterococcus faecalis* could secrete cytolysin, a biallelic toxin, which is released from the intestine into the portal vein and then into the liver, where it can directly perforate hepatocytes and induce death [76,77]. Duan et al. reported a strong correlation between cytolysin levels in AFLD patients and the severity of their liver injury and mortality [77]. When the phage treatment targeting *Enterococcus faecalis* was given in the intestine, it significantly reduced cytolysin levels in the liver and reduced the severity of ethanol-induced liver disease [77]. Meanwhile, in humanized mice colonized with fecal bacteria from AFLD patients, *Candida albicans* secreted candida hemolysin, which was also found to promote AFLD [78]. In a similar way to cytolytic hemolysin, candida hemolysin could damage hepatocytes and correlate with the severity of liver disease and mortality in AFLD patients [78].

*Lactobacillus* prevented AFLD by inhibiting oxidative stress, improving the function of the intestinal barrier, and reducing endotoxemia in the g intestine-liver axis [79]. The low number of Bacteroides and Firmicutes in the intestinal microbes of AFLD patients leads to dysbiosis of the intestinal ecology and overgrowth of pathogenic bacteria [80]. *Lactobacillus* could regulate the intestinal microbes of AFLD patients and normalize the intestinal microbes [81]. Previous studies have shown that *Levilactobacillus brevis* HY7410 and *Limosilactobacillus fermentum* MG590 reduced blood alcohol concentration by enhancing alcohol dehydrogenase and aldehyde dehydrogenase activity [81]. Many other studies have also found that *Limosilactobacillus reuteri* DSM17938, *L. brevis* SBC8803, and *L. fermentum* all alleviated alcohol-induced liver damage in mouse models [82,83].

## 6. Intestinal Microbes and Chronic Hepatitis B (CHB) and Chronic Hepatitis C (CHC)

### 6.1. CHB

Chronic hepatitis B (CHB)-induced liver damage is mediated by the immune response owing to the human hepatitis B virus. Hepatitis B virus infection remains a major public health problem affecting more than 250 million people worldwide [84]. In recent years, it has been reported that intestinal microbes interacted with infectious diseases such as enterovirus, influenza, and hepatitis B, and influenced host immunity [85]. Clinical studies have found a reduced proportion of Bifidobacteriaceae/Enterobacteriaceae in patients with CHB, with an increased abundance of Bifidobacteria and Lactobacillus and a reduced abundance of Enterococcus and Enterobacteriaceae [86]. The ratio of Firmicutes/Bacteroidetes not only impacts the carbohydrate metabolism of the body but is also considered to be an important factor associated with obesity and inflammatory processes [87]. Similarly, it has been demonstrated that the abundance of Bifidobacteria and Lactobacillus in the intestine of CHB patients was increased compared to healthy individuals, the abundance of Firmicutes was reduced and the ratio of Firmicutes/Bacteroidetes was increased which may accelerate the progression of cirrhosis and inflammation [88]. In addition, when intestinal permeability was altered, it was accompanied by bacterial translocation and the presence of portal vein endotoxins. This led to increased activation of TLR/ nucleotide-binding domain and leucine-rich repeat (NLR) in the liver, resulting in the production of cytokines that led to liver lesions, fibrosis progression, cirrhosis, and the development of hepatocellular carcinoma.

### 6.2. CHC

The hepatitis C virus infects 180 million people worldwide and over 4 million in the United States [89]. People with chronic hepatitis C (CHC) experience ongoing inflammation and progress to cirrhosis and the eventual development of liver disease [90]. Although inflammation is considered a normal defense mechanism against viral infection of hepatocytes, persistent unregulated inflammation is a major cause of chronic liver injury induced by hepatitis C virus infection [91]. According to published data, intestinal microbes found in patients with CHC showed a lower microbial diversity compared to healthy controls. This altered diversity also correlates directly with the severity and stage of CHC [92]. This CHC-associated reduction in intestinal microbe diversity was alleviated following antiviral drug treatment [93]. Further studies have found that Clostridiales are significantly reduced and both *Lactobacillus* and *Streptococcus* are significantly increased in the intestines of patients during hepatitis C virus infection [94]. There is also good evidence that the phylum of Bacterioidetes, the family of *Enterobacteriaceae*, and *Viridans streptococci* increased in abundance and the phylum of Firmicutes decreased in patients with chronic CHC [93]. LPS, a major component of gram-negative bacteria, promoted inflammation in the intestine when the intestine was damaged, and the inflammatory response in other organs of the body was further exacerbated when patients developed high blood LPS levels [95]. It was also noteworthy that there was an association between CHC and elevated LPS serum levels, suggesting that damage to the intestinal barrier as well as microbial dysbiosis and inflammatory responses play a non-negligible role in the progression of CHC [96].

Long before cirrhosis in patients with CHB and CHC, their fecal microbiota showed reduced diversity, elevated such as Enterobacteriaceae and Bacteroides, and elevated levels of other pathogens [93,97]. It has been found that interferon has a potential balancing effect in the intestine, by which pathogenic microbes can be eliminated and beneficial microbes can be promoted [98]. However, interferon therapy of patients with CHC cirrhosis showed that the microbes between pre- and post-treatment failed to induce microbe recovery [99]. Instead, antiviral drugs significantly improved the intestinal microbial composition in patients with CHC cirrhosis [100]. While the pathogenesis of cirrhosis was different. General microbial changes were critical in all of them. Currently, we need more data on hepatitis virus-associated liver diseases, especially the impact of anti-viral therapy on microbes.

## 7. Intestinal Microbes and Autoimmune Liver Disease (AIH)

The liver has been proposed as an innate immune organ because it is responsible for the production of most immune factors in the circulation and contains an abundance of resident innate immune cells [101]. Autoimmune hepatitis (AIH) is a complex chronic liver disease mediated by an excess of autoimmunity in the liver. AIH occurs in children and adults, predominantly in women, and the incidence of the disease may be increasing recently [102,103]. The mechanisms underlying the pathogenesis of autoimmune hepatitis have not been fully elucidated up to now. Genetic susceptibility, environmental factors, and immune tolerance breakdown are thought to be important factors in the development and progression of AIH [104]. Interestingly, some studies have reported a strong link between dysbiosis of intestinal microbes and AIH.

The early experiments discovered that germ-free mice were able to resist Concanavalin A (Con A)-induced acute AIH compared to normal mice [105]. Moreover, the administration of gentamicin attenuated Con A-induced AIH liver damage by depleting gram-negative bacteria of intestinal origin while reducing infiltration of liver immune cells, whereas the administration of exogenous pathogenic bacteria exacerbated Con A-induced acute hepatitis [106]. Furthermore, in a recent study, antibiotics-treated mice transplanted with the fecal microbes of mice exposed to trichloroethylene exhibited an AIH phenotype accompanied by an increase in systemic autoantibodies and increased liver inflammation compared to controls [107].

Wei et al. analyzed the fecal microbes composition of AIH patients and healthy controls and found that microbial dysbiosis in AIH patients was characterized by lower bacterial diversity and altered relative abundance, with the genus *Veillonella*, which is highly relevant to AIH, being highly enriched in AIH patients [104,107]. At the phylum level, Verrucomicrobia abundance was increased, while Lentisphaerae and Synergistetes were significantly decreased in the AIH patients [108]. At the same time, some studies have found a decrease in the relative abundance of *Bifidobacterium* in the feces of patients with autoimmune hepatitis [104]. Several studies have established AIH mouse models that almost mimic the condition of AIH patients and have analyzed the fecal microbes of these models. The results showed an increased abundance of *Proteobacteria* and *Bacteroidetes* at the gate level compared to controls, with the increase in Proteobacteria (facultative anaerobic bacteria) thought to be associated with inflammation, epithelial dysfunction, and disruption of the host-microbes balance [109]. A number of specific microbes have also been shown to correlate with the severity of AIH, for example, the abundance of *Veillonella* positively correlated with the degree of liver damage and the grade of inflammation in the liver [104]. The decline of *Bifidobacterium* was also associated with increased disease activity. In addition, an increase in LPS induced by AIH ecological dysregulation was shown to correlate with the advanced stages of the disease [110].

The intestinal microbes in the AIH disease model alter SCFAs as well as a range of metabolites including bile acids, which affect the integrity and permeability of the intestinal barrier and immune homeostasis. In models of AIH disease, a reduction in the anaerobic bacterium *Ruminococcus* leads to a reduction in SCFAs, which exacerbates the inflammatory response to AIH [108]. In AIH model mice, increasing the abundance of *Clostridium* with *Bifidobacterium animal lactic acid* 420 (B420) increased the levels of SCFAs and attenuated AIH-induced liver injury and intestinal barrier damage [111]. However, some studies have also shown that SCFAs can attenuate the inflammatory response to AIH mediated by lymphocytes by increasing Tregs cells and decreasing Th1 cells, suggesting that SCFAs in the liver may be able to attenuate the inflammatory damage of AIH [112]. The reduced abundance of *Clostridium* in AIH patients was also able to lead to a secondary decrease in bile acids and inhibit the polarization of natural killer T cells. Primarily through G protein-coupled bile acid receptor-1 (GPBAR1) inactivation, it promoted the secretion of the anti-inflammatory cytokine interleukin 10 (IL-10), thereby attenuating liver damage in Con A-induced hepatitis [113].

## 8. Intestinal Microbes and Hepatocellular Carcinoma (HCC)

HCC is one of the most common solid malignancies and is the leading cause of cancer-related deaths worldwide. HCC has the sixth-highest incidence and fourth-highest mortality rate worldwide [114]. The development of HCC is a complex process involving multiple risk factors. The previously mentioned NAFLD and NASH, AFLD, and liver diseases involved in CHB and CHC due to viral infections may all become further pathologic and irreversibly develop HCC. It has been found that a reduction in host microbes following intestinal bacterial clearance in mice significantly inhibits the tumorigenesis of diethylnitrosamine-induced HCC [115]. Consistent with this, smaller and fewer liver tumors have been reported in mice grown under germ-free conditions in the HCC model [116]. These studies suggested that intestinal microbes are associated with promoting liver tumorigenesis and tumor cell proliferation.

All the mechanisms mentioned in the previous section on intestinal microbes associated with liver diseases: disruption of the intestinal-to-barrier, endotoxemia, intestinal microbial dysbiosis, and immune regulation promote the development of HCC. The high levels of *E. coli* and other Gram-negative bacteria which were reported to be present in the intestine of HCC patients were associated with elevated serum levels of LPS in patients [117]. Activated hepatic stellate cells expressing TLR4 have been found to respond to low levels of LPS, which further promotes the development of fibrosis and cirrhosis [116]. The implications of TLR4 activation due to enterobacteria in the development of HCC have been investigated in animal models of HCC [116]. It has also been demonstrated that activation of TLR4 in hepatocytes by LPS in enterobacteria promotes the development of HCC by increasing cell proliferation and inhibiting apoptosis [118].

The clinical investigation found that *Oribacterium* and *Fusobacterium* were the most common bacteria isolated from the tongue swabs of HCC patients [119]. Analysis of clinical data found that tumor formation in HCC was associated with the presence of specific intestinal microorganisms *Bacteroides*, Lachnospiracea, Incertae sedis, and *Clostridium* XIVa, which were enriched in the feces of HCC patients [120]. On the other hand, *Lactobacillus*, *Bifidobacterium*, and *Enterococcus* were significantly reduced in the intestine of patients with hepatocellular carcinoma [121]. Dapito et al. also discovered that intestinal disinfection and TLR4 inactivation reduced HCC by 80% to 90% and could be used as a potential HCC prevention strategy [116]. Finally, an investigation of a rat model of HCC showed that probiotic fermented milk was able to slow the growth volume of tumors by 40% by reducing the expression of *C-myc*, *Bcl-2*, *Cyclin D1*, and *Rasp-21* [122]. Treatment with probiotics resulted in high levels of *Prevotella* and *Oscillibacter* in the fecal microbes of mice [122].

## 9. Discussion

Although there are many papers reported above, it is still a novel field in the crosslink of intestinal microbes and chronic liver diseases. In most cases, the causative relationship between the pathogenesis of different liver diseases and intestinal microbial changes is unclear. These changes in intestinal microbes can provide a reinforcing effect for the diseases, or they may be the adaptation in response to the variation of the internal and external environment. Consequently, in-depth explorations need to be implemented to reveal the mechanisms by which intestinal microbes regulate liver diseases. If this causative relationship is elucidated, variations in specific intestinal microbes will become a potential critical indicator for the clinical non-invasive diagnosis of liver diseases. Furthermore, the most difficult problem with intestinal microbes is that they can be influenced by species, gender, environment, dietary habits, and etiology, and the composition and content of intestinal microbes are always changing dynamically, which triggers variability and uncertainty in the study [123].

With the rapid development of sequencing technology, our understanding of intestine microbes has gradually deepened. With the help of these techniques, we can also further explore the inextricable connection between the intestine and the liver. At the same time, the maturity of technology and in-depth research have also given us a new direction for the problems of microbial treatment of chronic liver diseases. HTS (such as 16s rRNA sequencing, and metagenomics sequencing) has been widely used to explore the impact of intestinal microbes on health and disease [4,124]. HTS is the technological basis for modern research on intestinal microbial changes and liver diseases. It links microbial community signatures to phenotypes in chronic liver diseases. In order to explore the mechanism of action of intestinal microbes on the liver more comprehensively, many studies have combined the HTS results of microorganisms with liver metabolomics [125] or proteomics [126] for multi-omics combined analysis, and combined blood metabolomics and proteomics [127]. Complementary multi-omics approaches could provide functional information along with gene sequencing data, and link microbial community signatures to molecular mechanisms of chronic liver diseases [126,128,129]. HTS could also be combined with other techniques, such as mass spectrometry [128], high-content imaging [130], cell sorting, and other molecular or non-molecular techniques [131]. It is possible to gain an in-depth understanding of the classification of microbiota, the relationship between changes in the abundance of different populations, and genetic variation. It is also able to study the modification of some genes in specific cells in the liver by changes in microbial abundance in more detail, which is conducive to the exploration of molecular targets.

Currently, metagenomic sequencing technology has been widely used to explore the composition of complex microbial communities, including the human intestinal microbiome [50,124]. However, metagenomics was unable to resolve genomes at the level of individual strains. Most of the above studies could only roughly find or guess the influence of a certain type or phylum of bacteria on liver diseases through the commonly used sequencing techniques. Studies have confirmed that technology has been used in the study of complex microbial samples such as intestinal flora, and the emergence of single-cell RNA sequencing technology has analyzed gene expression profiles at the level of single strains [132]. Unraveling the genome at the single-strain level from the microbial community will greatly aid our understanding of intestinal microbial behavior and its impact on health. Single-cell sequencing of intestinal microbes from various chronic liver diseases will help find the key roles of certain strains in the pathogenesis of liver diseases.

The spatial transcriptome is a hot spot in current single-cell omics research. Conventional single-cell transcriptome sequencing couldn’t determine information about the original location of cells [133]. There have been many studies examining the interaction and communication between liver cells in various chronic liver diseases using spatial transcriptome technology [134,135]. Spatial transcriptomic technology is essential to enhance our understanding of the mechanisms of liver disease occurrence and development. If spatial transcriptome sequencing technology is applied to detect intestinal microbes in various chronic liver diseases, the dynamic changes and distribution of intestinal microbes, as well as the impact of interactions between microbes on the liver will become clearer.

Spatial transcriptome can reflect spatial relationships between cells, but cannot provide tissue-level insights [136]. Today, the combination of organoid research with organoids-on-a-chip research is expected to be a critical new technology for investigating intestinal microbes and chronic liver diseases [137,138,139]. The human intestine as well as liver organoids and their incorporation into more complex organoids-on-a-chip setups were developed [139,140]. Although the technology is still at a nascent stage, the field has already yielded some pivotal discoveries on the ways in which microbes contribute to health and disease [139,140,141]. Consequently, the combination of organoids and microbes will open new avenues for host-microbes co-metabolism research and can effectively address the major problems we have described above, such as highly variable microbes, unclear targets of metabolites, and unstable and prolonged clinical treatment of probiotics. These new technologies will greatly promote future work on intestinal microbes and chronic liver diseases.

A number of probiotics and prebiotics have also been described above for the treatment of chronic liver diseases. The clinical significance of probiotics in liver disease is more and more prevalent, with clinical and animal data indicating the therapeutic potential of probiotics in chronic liver diseases. However, there are still relatively few probiotics in clinical use, probably due to the following four points. (1) There are obvious differences between the mouse models and clinic patients. In particular human intestinal microbes are distinguished from mouse intestinal microbes, and some beneficial microbes in mice exhibit no effect in humans [142]. (2) The individual difference in probiotic treatment is notable, which has an inevitable impact on the efficacy of probiotics. (3) The time and dosage of probiotics clinical treatment need to be further explored [143]. (4) The efficacy of probiotics for chronic liver diseases is influenced by their activity. Probiotics tend to lose their activity during preservation [144]. Despite these limitations, many studies still bring the prospect of the modulation of intestinal probiotics in the treatment of chronic liver diseases [144].

These questions have led researchers to consider biotherapeutics that use microbially derived components and metabolites as an alternative to probiotics. We refer to them as prebiotics. Prebiotics are safer and more stable to use in the treatment of chronic liver disease patients [145,146]. Since the efficacy of prebiotics is not dependent on the viability of probiotics, this means that in the treatment of chronic liver diseases, they can be used in combination with antibacterial agents or bacteriophages to reduce the abundance of harmful bacteria and at the same time play the role of probiotics. In addition, prebiotics are less sensitive to environmental conditions, resulting in extended shelf life and the ability to be stored and shipped at ambient temperatures [38]. Prebiotics offers a new approach to the treatment of chronic liver diseases. However, there is not many prebiotics have been studied and used. Therefore, the method of effectively converting the functional components of probiotics into prebiotics needs to be further studied and explored. These studies all aim to provide more safe and more convenient prebiotic drugs for the clinical treatment of chronic liver diseases.

The development of new technologies such as 16s RNA sequencing, metagenomics, single-cell sequencing, and spatial transcriptomics has greatly expanded our knowledge of the microbial diversity and liver biological functions of intestinal microbes in various chronic liver diseases. A key next step in the study of intestinal microbes affecting chronic liver diseases is by combining microbiome data with experimental results. Focusing on the pathogenesis of chronic liver diseases, we will search for new key targets between the liver and intestine that affect the occurrence of the diseases. Meanwhile, key technologies such as organoids provide new insights into the clinical application of intestinal microbes and their bioactive metabolites in the diagnosis and treatment of chronic liver diseases.

## 10. Conclusions

With the advancement of technology, it has begun to appreciate the relationship among intestinal microbes, intestinal permeability, and liver diseases. We have summarized three main features of the interactions between the intestinal microbes and liver diseases: (1) Chronic liver diseases were associated with changes in microbial fractions. Many intestinal factors, such as intestinal microbes, bacterial composition, and intestinal bacterial metabolites, are deeply involved in liver homeostasis. (Table 1). (2) Chronic liver diseases were associated with intestinal and liver inflammation. Alterations in intestinal microbes tend to favor intestinal inflammation, while bacterial fragments and metabolites, in turn, contribute to inflammation and fibrosis responses in the liver via portal vein stimulation. (3) Chronic liver diseases were associated with intestinal permeability. Dysbiosis in the intestine and an elevated abundance of pathogenic microorganisms tend to disrupt the integrity of the intestinal epithelium. Bacteria and their metabolites can be allowed to enter the portal vein, ultimately stimulating injury that affects the liver. This review has focused on the specific microorganisms associated with the pathogenesis of various liver diseases. Changing the composition of intestinal microbes has also become a new strategy for chronic liver diseases. Numerous animal studies demonstrated the exciting possibilities of antibiotics, probiotics, prebiotics, and even phage interventions on intestinal microbes for the treatment of various liver diseases (Table 2).

We focus on the changes in intestinal microbes in chronic liver diseases and introduce the ameliorating effects of probiotics and their bioactive metabolites (prebiotics) in chronic liver diseases. There have been some clinical advances in microbial therapy in relation to chronic liver diseases, but the field is currently a novel and advanced field for both patients and clinicians. We need to use new technologies to support the study of the mechanism of intestinal microbes affecting chronic liver diseases and provide more reliable new ideas for clinical treatment.

## Figures and Tables

**Table 1:** Dysbiosis of intestine microbes in chronic liver diseases.

Disease	Dysbiosis of the Microbes	Metabolite Changes	References
Intestinal Leakage	Facultative anaerobes *↑*	LPS ↑ SCFAs ↓	[29]
			[30]
NAFLD	Firmicutes *↓*	ethanol ↑ SCFAs ↓	[50]
	*Bacteroides ↑*		[51]
	Proteobacteria ↑ Enterobacteriaceae ↑ *Escherichia ↑*		[52]
	*Coprococcus* ↓ *Ruminococcus gnavus ↓*		[53]
AFLD	Bacteroidaceae ↓ Prevotellaceae *↓*	cytolytic hemolysin ↑ candida hemolysin ↑	[75]
	*Veilonella* ↑ *Enterococcus faecalis ↑*		[76,77]
	*Candida albicans ↑*		[78]
CHB	*Bifidobacteria* ↑ *Enterococcus ↓*	hepatitis C virus ↑	[86]
	Firmicutes ↓ *Lactobacillus ↑*		[88]
CHC	Bacterioidetes ↑ Enterobacteriaceae ↑ *Viridans streptococci* ↑ Firmicutes ↓	hepatitis B virus ↑ LPS ↑	[93]
	*Lactobacillus* ↑ *Streptococcus* ↑ Clostridiales *↓*		[94]
AIH	*Veillonella ↑*	LPS ↑	[104,107]
	Verrucomicrobia *↑*		[108]
	Synergistetes ↓ Lentisphaerae *↓*		
	Proteobacteria ↑ Bacteroidetes *↑*		[109]
	*Bifidobacterium ↓*		[104,110]
HCC	*E. coli ↑*	LPS ↑	[117]
	*Oribacterium ↑*		[119]
	*Fusobacterium ↑*		
	*Bacteroides ↑*		[120]
	Lachnospiracea *↑*		
	Incertae Sedis *↑*		

LPS, lipopolysaccharides; SCFA, short-chain fatty acids; NAFLD, non-alcoholic fatty liver disease; AFLD, alcoholic fatty liver disease; CHB, chronic hepatitis B; CHC, chronic hepatitis C; AIH, autoimmune liver disease; HCC, hepatocellular carcinoma. “↑” represents the increased abundance of the corresponding bacteria. “↓” represents the decreased abundance of the corresponding bacteria.

**Table 2:** Probiotics and prebiotics for the treatment of chronic liver diseases.

Disease	Probiotics	Prebiotics	References
Intestinal Leakage	*Lactobacillus, Bifidobacterium and Saccharomyces*	SCFAs, Insulin	[35]
	*Lactobacillus plantarum* MB452		[36]
	*Lactobacillus rhamnosus* GG (LGG)		[37]
	*Eubacterium rectale*, *Clostridium coccoides*, *and Roseburia*		[39]
NAFLD	VSL #3: *Streptococcus thermophilus*, *Bifidobacterium breve*, *Bifidobacterium longum, Bifidobacterium infantis*, *Lactobacillus acidophilus*, *Lactobacillus plantarum, Lactobacillus paracasei*, *and Lactobacillus bulgaricus.*	CLA	[60,61]
AFLD	*Lactobacillus*	Lactic acid	[79,81]
	*Levilactobacillus brevis* HY7410 and *Limosilactobacillus fermentum* MG590		[81]
	*Limosilactobacillus reuteri* DSM17938, *L. brevis* SBC8803, and *L. fermentum*		[82,83]
AIH	*Clostridium*	Antibiotics SCFAs Bile Acid	[113]
	*Clostridium with Bifidobacterium animal lactic acid* 420 (B420)		[111]
HCC	*Prevotella and Oscillibacter*		[122]

SCFAs, short-chain fatty acids; CLA, conjugated linoleic acid; NAFLD, non-alcoholic fatty liver disease; AFLD, alcoholic fatty liver disease; CHB, chronic hepatitis B; CHC, chronic hepatitis C; AIH, autoimmune liver; HCC, hepatocellular carcinoma.

## Data Availability

Not applicable.
